# Therapeutic Management of Children with Vesicoureteral Reflux

**DOI:** 10.3390/jcm13010244

**Published:** 2023-12-31

**Authors:** Valeria Chirico, Filippo Tripodi, Antonio Lacquaniti, Paolo Monardo, Giovanni Conti, Giorgio Ascenti, Roberto Chimenz

**Affiliations:** 1 Pediatric Nephrology and Dialysis Unit, University Hospital “G. Martino”, 98124 Messina, Italyfilippo.tripodi@opbg.net (F.T.);; 2 Nephrology and Dialysis Unit, Papardo Hospital, 98158 Messina, Italypmonardo66@gmail.com (P.M.); 3 Section of Radiological Sciences, Department of Biomedical Sciences and Morphological and Functional Imaging, University Hospital “G. Martino”, 98124 Messina, Italy

**Keywords:** contrast-enhanced voiding urosonography (ceVUS), vesicoureteral reflux (VUR), continuous antibiotic prophylaxis (CAP), febrile urinary tract infection (UTI), renal scars

## Abstract

Contrasting data refer to therapies for vesicoureteral reflux (VUR), such as surgical treatments and continuous antibiotic prophylaxis (CAP). This study evaluated the effectiveness of these approaches in children with VUR, analyzing the recurrence of febrile urinary tract infections (UTIs) and the resolution of VUR after the treatment. A total of 350 pediatric patients underwent contrast-enhanced voiding urosonography (ceVUS) to diagnose a VUR, whereas renal scintigraphy evaluated potential scars. After 12 months from the treatment, the VUR, the relapse of febrile UTIs, and reflux-related nephropathy were analyzed. Twenty-seven children had recurrent febrile UTIs after surgical therapy, with a greater rate of relapses observed in III and V VUR grades. Thirteen patients who underwent surgery had scars, independently of VUR grades and gender, with evidence of chronic renal failure at the end of the follow-up period. A total of 140 subjects were treated with CAP, and 30% of them continued to suffer from febrile UTIs. Ninety-five patients with VUR underwent ceVUS after 12 months, with persistent reflux in fifty-two patients. All of them had severe VUR, correlating with the age at diagnosis and gender. CAP therapy prevented scarring better than surgery, especially in children with III and V grades of VUR. A late onset of VUR or VUR involving neonatal patients is rarely a reversible process. This study identified predictors of success or failure of surgical or CAP therapies, evaluating the relapse of UTIs or persistent reflux after the treatment and giving prognostic information in children with VUR.

## 1. Introduction

Vesicoureteral reflux (VUR) is a topical issue without a total agreement for its management, essential for ensuring the healthy growth of children and improved quality of life [1]. It consists of an abnormal backward flow of urine from the bladder up to the kidneys, secondary to a structural or functional abnormality of the vesicoureteral junction or pathological increased intravesical pressure, due to neurogenic bladder, posterior urethral valves, or other obstructive uropathies [2].

Epidemiological data on VUR prevalence highlight a public health issue, rather than an area of expertise in pediatric nephrology. The mean prevalence of VUR is 1%, ranging from 0.4 to 2%, similar to other clinical conditions, such as celiac disease or cardiac malformations, and underestimated due to asymptomatic cases related to spontaneous resolution of VUR [3,4]. 

VUR affects males more than females, earlier and with higher severity, whereas females are affected at a later age with mild severity of RVU and with less probability of spontaneous resolution [5,6]. VUR, affecting 30% of children with a history of urinary tract infections (UTIs) and 17% with normal kidneys, should be suspected when recurrent febrile UTIs and lower urinary tract symptoms (LUTS) occur, whether or not they are associated with bladder bowel disorders (BBD) [7,8,9].

According to the Italian Society of Pediatric Nephrology (SiNePe), VUR represents a risk factor for UTI development [10], distinguishing two different entities related to it, “reflux disease” and “reflux symptom”; the first involves predominantly males, with a rare incidence, prenatally or under two years of age, with severe VUR (stage IV–V), abnormal renal parenchyma and urinary tract, and spontaneous resolution in 50% of cases. The second and more common form of VUR is usually assessed in females, with low-grade I–III VUR, associated with normal kidneys and urinary tract, with a high rate of resolution (80–90%) [11,12]. A voiding cystourethrogram (VCUG) is the “gold standard” for VUR detection, allowing grading of the severity from a wisp of contrast just beyond the bladder with no dilatation of the ureter or collecting system (grade I) up to dilatation and tortuosity of the ureters with calyceal clubbing (grade V). This radiologic test is an invasive procedure requiring urethral catheterization, often painful and traumatic for the child, causing UTIs in 1 to 3% of cases [13,14]. Although a diagnostic study can be achieved with a relatively low radiation dose by using careful technique and modern equipment, in practice, the range of doses is extremely wide [15]. 

Other tests have been proposed to detect reflux, such as contrast-enhanced voiding urosonography (ceVUS), an ionizing radiation-free technique using ultrasound with a contrast agent instilled into the bladder to image the urinary tract. Several studies revealed ceVUS as a valid alternative method for VUR assessment, with comparable results in terms of sensitivity and specificity with a VCUG in detecting and grading VUR. However, although ceVUS has several advantages, its limitations should be underlined, highlighting the lack of uniform standardization of the method, and some issues regarding ultrasound, such as bowel gas interference. The biggest limitation of ceVUS is the incapability to visualize the urethra and, in consequence, its low diagnostic ability to rule out congenital urethral pathologies, such as urethral valves. In this case, a VCUG is reserved for the correct diagnosis [16,17].

Behind radiological procedures, several urinary biomarkers are being studied to achieve early diagnosis, facilitating staging and therapeutic VUR management. In particular, some interleukins or neutrophil gelatinase-associated lipocalin have been associated with the innate immune reaction and proinflammatory state characterizing children with VUR, with potential clinical application to easily identify patients who require antibiotic prophylaxis or surgical intervention [18]. 

However, prospective and larger studies are needed to confirm the role of these or other biomarkers as alternative, non-invasive tools to VCUG and ceVUS.

The choices of the patients and the radiological test to perform are not unique challenges, considering that the therapeutic approach of VUR is a matter of debate. 

There are two distinct directions in the literature regarding the investigation of an uncomplicated first febrile UTI in a child. In general, when presented with a first febrile UTI in a child, physicians recommend fewer investigations and less treatment, in contrast to surgeons who advocate extensive investigation and aggressive intervention if imaging detects an abnormality [19].

Moreover, the child affected by VUR undergoes renal scintigraphy to complete the diagnostic process, evaluating the function of the kidneys. The risk of a child without other congenital abnormalities of the kidney and urinary tract developing chronic kidney disease as a result of repeated febrile UTIs associated with VUR is very low [20].

Spontaneous resolution of VUR can be observed in about more than 80% of grades I and II, around 45% of grade III, and less than 10% of grades IV and V [21]. 

According to the main international guidelines, VUR therapy is based on three strategies, depending on the severity of VUR and physicians’ preferences [22]. 

In children without UTI symptoms and with low grades of VUR, the “wait and watch” approach could be considered due to the high probability of spontaneous resolution [23]. 

If it is known that VUR could resolve spontaneously over time, waiting for this to occur rather than treating it should only happen in the absence of repeated febrile UTIs, a risk factor for renal scarring [24].

Moreover, VUR and BBD are closely related and around half of patients with VUR also have BBD. In this context, effective treatment of BBD can increase the chance of spontaneous resolution of VUR [25].

However, regular follow-up visits are required to enable adequate monitoring of the patient’s status, and this approach is recommended for patients with a relatively low risk of renal injury, such as males with low-grade VUR.

Conversely, independently from the severity of the VUR, in children with LUTS and recurrent UTIs, continuous antibiotic prophylaxis (CAP) could be prescribed [26]. 

However, randomized trials failed to establish a precise identikit of a child requiring a CAP strategy, suggesting an accurate identification of the risks, personalization of the treatment, and the correct timing of treatment to achieve efficient results [27,28,29]. 

Misuse of antibiotics increases the spread of antimicrobial resistance in community-acquired UTIs, which is already an alarming threat further limiting the effectiveness of available antibiotics [30].

Moreover, an altered gut microbiota composition characterized infants with VUR previously treated with CAP, even after a short exposure to a sub-therapeutic dosage of antibiotics, predisposing them to high amounts of Enterobacteriaceae, such as Klebsiella and *Escherichia coli* spp., representing a non-negligible risk factor for the development of more difficult-to-treat UTIs. Adverse effects of long-term antibiotic use such as allergic reactions, weakened immune system, and *Clostridium difficile* infection should also be considered [31].

Lastly, endoscopic and surgical procedures represent alternative options, considering the high rate of resolution of VUR, but there are contrasting data about the incidence of UTI- and VUR-related nephropathy after the procedures [32]. 

Ureteral re-implantation has been the main surgical technique used, with successful outcomes for VUR correction, by creating a longer ureteral segment, passing the tunnel between the bladder mucosa and muscularis propria, miming the anti-reflux mechanism. The Lich–Gregoir extravesical anti-reflux technique, Cohen intravesical reimplantation, and the Politano–Leadbetter combined intravesical and extravesical reimplantation technique are the most commonly used methods [33].

Moreover, recent data related to minimally invasive surgery, supported by the use of robot-assisted surgery for the management of VUR, revealed a reduction in side effects compared to open surgery [34]. However, more consolidated data and longer follow-ups are needed to corroborate these results.

Endoscopic injection has reported widespread use in the last two decades for VUR treatment, and it is considered a minimally invasive technique, performed on an outpatient basis, and with a relatively short learning curve and low complication rate. The subureteric injection of bulking material, such as dextranomer/hyaluronic acid copolymer and polyalcohol polyacrylate copolymer, supports the submucosal tunnel in its intramural path. However, endoscopic treatment may also have complications, i.e., ureteral obstruction [35,36].

According to these data, surgical correction, not modifying the natural history of the disease, is proposed when CAP fails, especially in females with high grades of VUR [37]. While the European Pediatric Urology guidelines recommend surgical correction of high-grade VUR with uretero-vesical re-implantation, basing these suggestions on non-comparative observational studies over two decades old, recent clinical trials comparing endoscopic management vs. uretero-vesical re-implantation in high-grade VUR support the use of endoscopic management as a therapeutic option [6,38].

Moreover, the 2019 Cochrane Review reported that despite a significant reduction in repeat episodes of febrile UTIs reported after surgery, there were no differences between surgery and CAP use in either symptomatic UTIs or renal damage. However, correcting VUR using endoscopic approaches would theoretically reduce the risk of adverse events associated with surgery [39].

Starting from these assumptions, this study evaluated the effectiveness of CAP and endoscopic approaches in children with VUR, analyzing, as the endpoints, the recurrence of febrile UTIs and the resolution of VUR after the treatments.

## 2. Materials and Methods

### 2.1. Patient and Study Design

A total of 350 pediatric patients, followed at the Pediatric Nephrology and Dialysis Unit of the “G. Martino” University Hospital of Messina, were enrolled retrospectively between September 1999 and March 2023. ceVUS was conducted to diagnose RVU in children with ultrasound abnormalities, such as congenital anomalies of the kidney and urinary tract (CAKUT) and/or pelvicalyceal dilatation, LUTS, and febrile UTIs, associated with positive urine cultures with significant bacterial growth defined as >100.000 colony-forming units. 

Before the injection of the ultrasound contrast agent SonoVue, 5 mL of sterile physiological saline was injected into the contrast agent, the mixture was shaken until the freeze-dried powder was completely dissolved, and 0.5 mL of microbubble suspension was obtained for further use. We assessed reflux in real time by scanning the bladder, ureter, and renal pelvis. After using the contrast agent, we drained the contrast agent from the urinary tract and removed the catheter. Adverse reactions throughout and after the examination were then examined and recorded. All examinations were performed by the same senior physician (G.A. in the author list) who had experience in imaging techniques.

According to the SiNePe criteria, the first UTI onset established three classes of patients: within 28 days of birth, between 28 days and 2 years, and over 2 years [40], whereas BBD patients have been identified according to the standardization committee of the International Children’s Continence Society [41]. 

VUR was stratified into mild (stages 1–2), moderate (stage 3), and severe (stages 4–5) according to the classification system proposed by the International Committee on Reflux Research currently used for traditional VCUG. In particular, grade I: reflux reaches the ureter; grade II: reflux into the ureter, renal pelvis, and renal calyces but without any dilatation; grade III: reflux with mild or moderate dilatation of the renal pelvis but no or only mild dullness of the renal calyces; grade IV: moderate dilatation of the renal pelvis or moderate distortion of the ureter, with complete disappearance of the acute angle of the renal calyces, which mostly remain papillary depressed; grade V: considerable dilatation of the renal pelvis and calyces; the ureter is greatly dilated and distorted, and most of the renal calyces have lost their nipple shape [42].

According to the therapeutic strategies, patients were divided into two groups: the CAP group included VUR patients treated with antibiotics, whereas patients treated by endoscopic procedures characterized the surgical group. Patients who failed a CAP approach and were then treated by endoscopic surgery were excluded from the study. 

Hemodialyzed children or those with renal transplants were excluded from the study. Moreover, all male patients with RVU diagnosed by ceVUS underwent VCUG to diagnose posterior urethral valves. These patients were excluded from the study. 

### 2.2. Outcomes and Follow-Up

In VUR patients, we revealed the main characteristics of the reflux, such as the laterality, the severity, and the age at diagnosis. Six months after the diagnosis of VUR, renal dimercaptosuccinic acid (DMSA) scintigraphy was performed to evaluate the function of the kidneys and potential scars. We also divided the VUR cohort according to the type of treatment, evaluating the duration of the therapy and the type of antibiotic in the CAP group. In the treated group, we analyzed post-surgery and post-CAP breakthrough infections. After 12 months from the treatment, a ceVUS indicated the presence or lack of VUR, its severity, and grades. 

**Figure 1:**
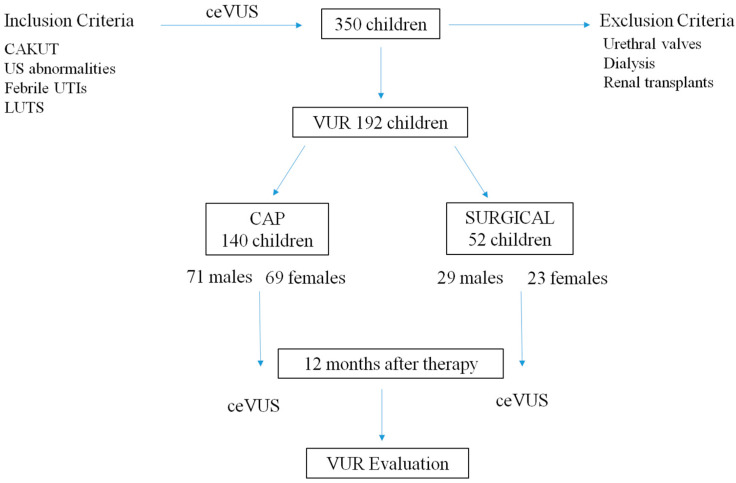
Inclusion and exclusion criteria and therapeutic strategies. Abbreviations: CAKUT: congenital anomalies of the kidney and urinary tract; IS: ultrasound; UTIs: urinary tract infections; LUTS: lower urinary tract symptoms; ceVUS: contrast-enhanced voiding urosonography; VCUG: voiding cystourethrogram; VUR: vesicoureteral reflux; CAP: continuous antibiotic prophylaxis.

Figure 1 summarizes the inclusion and exclusion criteria, and the division into two groups according to the therapeutic strategies.

### 2.3. Statistical Analyses

Statistical analyses were performed with NCSS for Windows (version 4.0), MedCalc (version 11.0; MedCalc Software Acacialaan, Ostend, Belgium) software, and the GraphPad Prism (version 5.0; GraphPad Software, Inc., San Diego, CA, USA) package. Data were presented as mean ± SD for normally distributed values (from the Kolmogorov–Smirnov test) and median [IQ range] for non-normally distributed values.

Differences between groups were established by an unpaired t-test or by ANOVA followed by Bonferroni’s test for normally distributed values and by Kruskal–Wallis analysis followed by Dunn’s test for nonparametric values. All results were considered significant if *p* was <0.05.

## 3. Results

### 3.1. Epidemiologic Data

We enrolled 350 patients, of whom 170 were female (48.6%) and 180 male (51.4%); of them, 192 children had a VUR, diagnosed more in males than females (52% vs. 47%; *p* < 0.05). Moreover, male children had a greater severity of VUR (grade II–III: 43%; grade IV–V: 57%) compared to females (grade II–III: 60%; grade IV–V: 40%).

VUR severity was not related to the age at diagnosis in males who were affected within the first two years of age. Moreover, a precocious diagnosis was achieved only in males during the neonatal period, whereas the first diagnosis of VUR was assessed in females later, after two years of age. All children who underwent treatment were divided into two groups, according to the type of treatment. 

### 3.2. Surgical Approach

**Figure 2:**
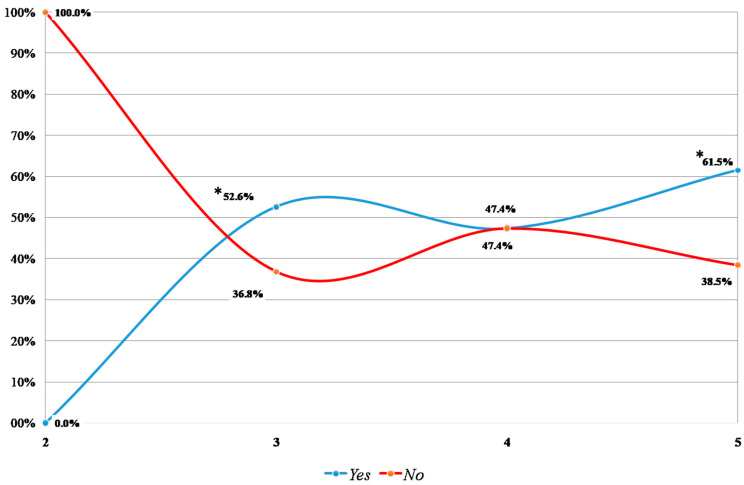
Febrile UTI relapse after surgical therapy according to VUR grades. *: *p* < 0.05.

A total of 52 patients (29 M and 23 F) underwent endoscopic surgical therapy. Twenty-seven children had recurrent febrile UTIs after the surgical treatment, with similar involvement in females (n: 14, 51%) and males (n: 13, 49%). Moreover, BBD was diagnosed more in females (n: 11, 79%) than in males (n: 6; 46%). All these BBD patients underwent endoscopic treatment after the failure of medical therapies based on anticholinergic drugs, i.e., oxybutynin, and behavior modifications. A greater rate of febrile UTI relapse was observed in children with III and V VUR grades (Figure 2).

In this cohort, we revealed a direct relation between recurrent febrile UTIs and positive urine cultures, characterizing 92% of subjects (25 children out of 27). Moreover, 13 patients who underwent surgery showed scars in renal scintigraphy, independently of VUR grades and gender. These children were younger, with atypical bacteria in the urine cultures and with CAKUT. 

### 3.3. Continuous Antibiotic Prophylaxis (CAP)

A total of 140 subjects were treated with CAP, without differences between males (n: 71) and females (n: 69). Forty-two of them (30%) had febrile UTIs after the end of the antibiotic therapy, with a greater incidence in females than males. In particular, breakthrough UTIs affected 40% of females, whereas relapses of UTIs were assessed in 20% of males (*p* < 0.05). According to the severity of the reflux, as observed in the surgical group, we observed more episodes of UTIs in patients with grades III and V, both in males and in females. In the latter, a higher diagnosis of BBD was recorded, associated with a higher number of renal scars, compared to males. According to the age of the patients, children aged >2 years had a higher incidence of febrile UTI relapse than younger patients (38.5% vs. 29.9%; *p* < 0.05). Moreover, patients with positive urine cultures before CAP therapy had a higher risk of relapse of UTIs (38 patients, 90% vs. 5.10%; *p* < 0.05). 

**Figure 3:**
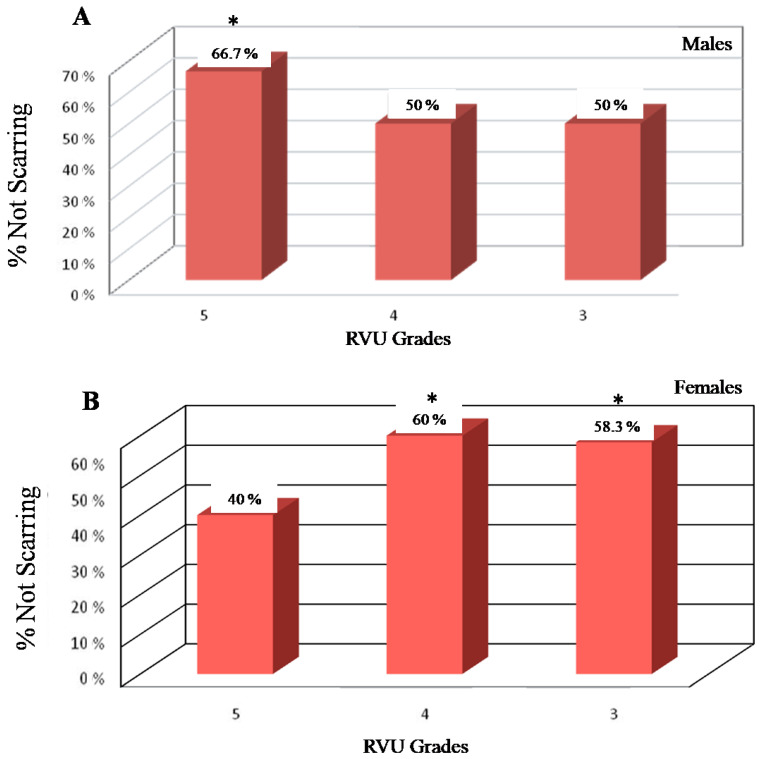
CAP therapy and renal scarring during the follow-up period. *: *p* < 0.05. Males had renal scarring prevention in grade V of VUR (**A**), whereas females with grades III and IV of VUR obtained more scarring prevention (**B**) after CAP therapy.

Furthermore, CAP therapy did not solve the renal scarring during the follow-up period, but it prevented the scars better than surgery, especially in males with grade V of VUR, and in females with III and IV grades of VUR (Figure 3).

### 3.4. VUR and ceVUS after 12 Months

**Figure 4:**
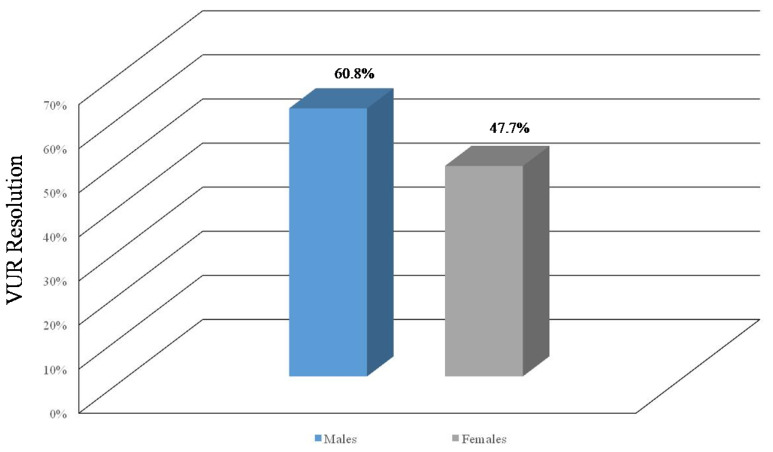
Persistent reflux after a follow-up of 12 months.

A total of 95 patients with VUR underwent ceVUS after 12 months to evaluate the resolution of the reflux. We demonstrated that the reflux persisted in patients with severe VUR, with a correlation between the age at diagnosis and gender, behind the severity of the reflux. In our cohort, 60% of males (31 out of 51; *p* < 0.05) had persistent reflux, whereas this condition was revealed in 21 females (21 out of 44, 47%; *p* < 0.05) (Figure 4).

Conversely, the resolution of VUR was diagnosed in children between 28 days and 2 years of age, revealing that a late onset of VUR or VUR involving neonatal patients rarely is a reversible process.

## 4. Discussion

**Figure 5:**
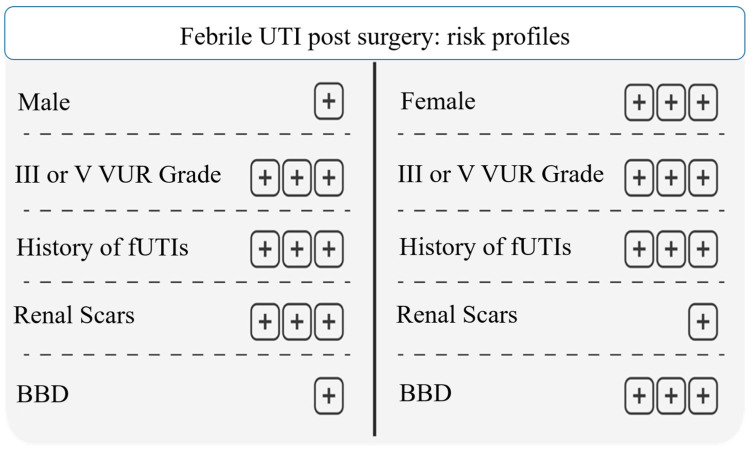
Febrile UTI post surgery: risk profiles. Abbreviations: UTI: urinary tract infection; VUR: vesicoureteral reflux; fUTI: febrile urinary tract infection; BBD: bladder and bowel dysfunction.

This study identified different risk profiles to justify the choice of VUR treatment, expecting a specific result after surgical endoscopy or CAP therapies; the efficacy of the therapy was identified by the prevention of febrile UTI after the treatment of VUR and/or the resolution of the reflux assessed by ceVUS. Our data revealed that 50% of surgical patients had a relapse of UTIs after the treatment. However, by analyzing our cohort, we could define a patient profile, predicting a high risk of surgical failure to solve VUR. According to our data, male and female patients with grade III or V VUR, with recurrent febrile UTIs and renal scars, have a higher risk of surgical failure, not solving VUR, or not preventing febrile UTIs. The diagnosis of concomitant BBD does not change the results in males, although it must always be treated, whereas in females the presence of LUTS and/or BBD increases the risk of therapy failure (Figure 5).

Conversely, patients with grade IV VUR had a better prognostic profile, with less incidence of febrile UTI relapse and renal scars after the surgical approach, suggesting a potential surgical approach in these patients if CAP therapy fails. 

Reflux-related nephropathy represents the most feared complication of VUR, causing a chronic inflammatory disease with progressive loss of GFR [43]. This evolution does not affect all VUR patients, but it interests only patients with genetic predisposing factors, and if associated with febrile UTIs, it determines the progression of kidney disease until the start of renal replacement therapy [5,6,44]. 

Whether VUR contributes to the risk of chronic kidney disease (CKD) remained unclear. Several pathophysiological mechanisms could cause CKD development in pediatric VUR patients, through parenchymal thinning and atrophy until fibrosis with subsequent decreased renal function secondary to acute pyelonephritis and urine retention [45].

All these processes involve a developing kidney, leading to an interruption of glomerular maturation, necrosis, apoptosis of tubular cells, and interstitial inflammation [46].

The first prospective evaluation of the incidence of CKD in children and adolescents with VUR was obtained by a prospective Italian population-based registry, named ItalKid Project, in the 1990s [47]. 

Recently, a population-based cohort study revealed a 3.78-fold increased risk of CKD in a VUR cohort compared with a non-VUR cohort in children aged >1 year [48].

However, further trials are required to evaluate the correlation between VUR and CKD in the pediatric population, as well as the role of precocious therapies in preventing these complications. In particular, the therapeutic strategy for VUR is a debated topic. Several data highlighted that corrective endoscopic treatments did not reduce the risks of febrile UTIs and renal scars, whereas other results assessed the contrary, suggesting them as first-line therapy to manage a patient with VUR [11,49,50,51].

In recent decades, CAP therapy has been progressively diminished, moving from being the first therapeutic option in all patients with VUR, regardless of the degree, to being indicated exclusively in subjects with grades III–V VUR for 1–2 years. Several studies analyzed the role of CAP in children, with contrasting data about its effectiveness in modifying the natural history of VUR and UTI relapses and preventing kidney scars [52,53,54]. 

Our data underlined that 33% of patients had febrile UTIs after CAP, not eliminating them but reducing the risk compared to the endoscopic approach. A recent randomized trial supports these considerations underlining that in VUR infants with grade III–V and no previous UTIs, CAP provided a small but significant benefit in preventing UTIs [55]. 

According to the data obtained from the CAP group, we underlined the profile of VUR patients characterized by a higher risk of treatment failure. Similar to the surgical group, grades III and V were related with the worst prognosis, compared to grade IV, with particular attention on patients with recurrent UTIs before CAP therapy and BBD. Our data confirm that renal scars represent a risk factor for febrile UTIs, with a higher risk assessed in males than females [56]. However, renal scars must be related to clinical information and prognostic markers, such as proteinuria, blood hypertension, or a progressive reduction in the glomerular filtration rate.

**Figure 6:**
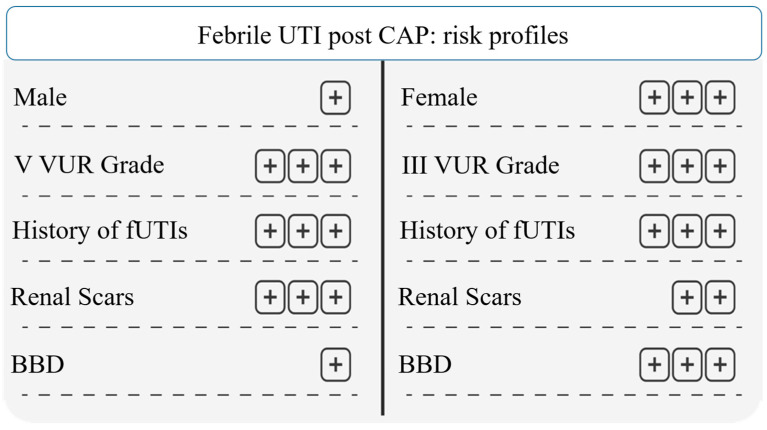
Febrile UTI post CAP: risk profiles. Abbreviations: UTI: urinary tract infection; VUR: vesicoureteral reflux; fUTI: febrile urinary tract infection; BBD: bladder and bowel dysfunction.

This study underlines that the routine use of CAP should be personalized and not administered in all VUR patients, avoiding the routine use of the CAP strategy, and allowing for a reduced risk of antibiotic resistance and adverse effects on gut microbiota (Figure 6).

The personalized CAP approach suggested by our study strengthens the results obtained by cost-effectiveness analyses, such as those underlined by the RIVUR trial, which revealed that the CAP strategy was only cost-effective if administered to patients with grade IV VUR whereas costs per quality-adjusted life year gained in patients with grades I–III VUR were deemed prohibitively high [57,58].

The retrospective analysis and the relatively small number of enrolled patients represent the main study limitation. However, we included participants with similar characteristics in both treatment groups, and the efficacy of CAP and surgery revealed a real-life scenario in which the antibiotic therapy, chosen according to local patterns of bacterial resistance, could represent the common clinical practice and allow for generalizing our results to all VUR children.

## 5. Conclusions

This study identified predictors of success or failure after surgical and/or CAP approaches, evaluating the relapse of UTIs or persistent reflux after treatment, and giving prognostic information in children with VUR. The risk stratification in VUR children allows for personalized therapies, avoiding overtreatment in childhood and reducing several adverse effects, such as antibiotic resistance or complications related to surgical procedures. 

## Data Availability

The dataset generated and analyzed during the current study is available from the corresponding author upon reasonable request.
